# Clinicopathological Challenge: A New Article Type in *Dermatopathology*

**DOI:** 10.3390/dermatopathology11030025

**Published:** 2024-08-14

**Authors:** Gürkan Kaya

**Affiliations:** 1 Department of Dermatology, University Hospital of Geneva, 1205 Geneva, Switzerland; gkaya@hug.ch; 2 Department of Clinical Pathology, University Hospital of Geneva, 1205 Geneva, Switzerland

As the Editor-in-Chief of *Dermatopathology*, I have the great pleasure of announcing a new article type: “Clinicopathological Challenge”. As indicated in the Manuscript Submission Overview in the section Instructions for Authors, this new article type will present and discuss challenging clinical cases supported by clinical and histopathological photographs. A clinicopathological challenge article will include an abstract, keywords, and four sections entitled “Case Presentation”, “What Is the Diagnosis?”, “Diagnosis”, and “Discussion”. The “What Is the Diagnosis?” section will discuss five possible diagnosis options. The included figures will contain 2–3 unpublished clinical and histopathological photographs. After submission, clinicopathological challenge manuscripts will undergo the same peer review process as a regular research article.

The clinicopathological challenge will join the existing article formats: article, review, and case report. The format organized in the form of a quiz will allow readers to test themselves by examining the clinical and histopathological information, going through the proposed multiple-choice questions, and comparing the different diagnoses according to a clinicopathological correlation. I think that this format will be very instructive and have high didactic value for both novice and experienced dermatopathologists.

I would like to invite all potential authors to submit manuscripts adapted for this new format, which I believe will be beneficial for the readers of *Dermatopathology*.

